# Differences in Tissue Copper and Zinc Content Between Normal Livers and Those with Cirrhosis with or Without Hepatocellular Carcinoma

**DOI:** 10.3390/ijms26146571

**Published:** 2025-07-08

**Authors:** Simona Parisse, Giulia Andreani, Monica Mischitelli, Alessandra Gianoncelli, Emil Malucelli, Michela Fratini, Flaminia Ferri, Maria Carlucci, Quirino Lai, Andrea Ascione, Gianluca Mennini, Massimo Rossi, Stefano Iotti, Gloria Isani, Stefano Ginanni Corradini

**Affiliations:** 1 Department of Translational and Precision Medicine, “Sapienza” University of Rome, Viale dell’Università 37, 00185 Rome, Italy; simona.parisse@uniroma1.it (S.P.); monica.mischitelli@uniroma1.it (M.M.); flaminia.ferri@uniroma1.it (F.F.); maria.carlucci@uniroma1.it (M.C.); 2 Department of Veterinary Medical Sciences, Alma Mater Studiorum-University of Bologna, Via Tolara di Sopra 50, 40064 Ozzano dell’Emilia, Bologna, Italy; giulia.andreani2@unibo.it; 3 Elettra-Sincrotrone Trieste, Strada Statale 14 km 163,5 in AREA Science Park, Basovizza, 34149 Trieste, Italy; alessandra.gianoncelli@elettra.eu; 4 Department of Pharmacy and Biotechnology, University of Bologna, 40126 Bologna, Italy; emil.malucelli@unibo.it (E.M.); stefano.iotti@unibo.it (S.I.); 5 Department of Physics, CNR-Nanotec (Rome Unit) c/o, Sapienza University of Rome, Piazzale Aldo Moro 7, 00185 Rome, Italy; michela.fratini@gmail.com; 6 Laboratory of Neurophysics and Neuroimaging (NaN), IRCCS Fondazione Santa Lucia, Via Ardeatina 306, 00179 Rome, Italy; 7 General Surgery and Organ Transplantation Unit, “Sapienza” University of Rome, Viale del Policlinico 155, 00161 Rome, Italy; quirino.lai@uniroma1.it (Q.L.); gianluca.mennini@uniroma1.it (G.M.); massimo.rossi@uniroma1.it (M.R.); 8 Department of Experimental Medicine, “Sapienza” University of Rome, Viale Regina Elena 324, 00161 Rome, Italy; 9 National Institute of Biostructures and Biosystems, Via delle Medaglie d’oro, 305, 00136 Rome, Italy

**Keywords:** cirrhosis, atomic absorption, hepatocellular carcinoma, cancer, liver, copper, zinc, magnesium, metals, liver transplant

## Abstract

This study aimed to compare the contents of copper (Cu), zinc (Zn), magnesium (Mg), and iron (Fe) in healthy liver tissue from deceased liver donors (DGs), in cirrhotic tissue from patients without (CIR) or with hepatocellular carcinoma (CIR-HCC) and in HCC tissue from the latter patients. Liver tissue samples were obtained from cirrhotic liver transplant recipients, with (*n* = 14) and without HCC (*n* = 14), and from DGs (*n* = 18). In patients with HCC, both cirrhotic and tumor tissue was collected. The tissue metal content was measured using atomic absorption spectrometry. The Cu content of DG tissue was significantly lower than that of CIR-HCC and HCC tissue but not CIR tissue. The tissue Zn and Mg contents were significantly higher in DG tissue than in CIR, CIR-HCC, and HCC tissues. No difference was observed for Fe. The Cu/Zn ratio progressively increased in DG, CIR, CIR-HCC, and HCC tissues. The increased Cu content in cirrhotic and tumor tissue of HCC patients and the fact that the latter had the highest value for the Cu/Zn ratio indirectly suggest the potential role of these metals in hepatocarcinogenesis. These findings support a pathophysiological basis for further experimental studies to investigate the potential therapeutic implications of pharmacological agents targeting metal homeostasis in this malignancy.

## 1. Introduction

In hepatology, the relationship between various metals in the serum and/or liver tissue and hepatocellular carcinoma (HCC) is intriguing and has been frequently debated.

Copper (Cu), zinc (Zn), magnesium (Mg), and iron (Fe) are metals involved in cellular homeostasis. Furthermore, an increase or decrease in the cellular content of these metals has been described as a cause or a contributing factor in various diseases and types of cancer, including skin, gastrointestinal tract, lung, breast, and prostate cancers [1,2,3,4,5,6,7].

As an essential trace element, Cu is physiologically involved in various cellular processes, including antioxidant defenses, as a cofactor of superoxide dismutase, oxidative stress, protein synthesis, and energy metabolism as a cofactor of cytochrome oxidase [8].

However, given its broad spectrum of biological activity, Cu may play an ambivalent role and its potential involvement in hepatocellular carcinoma (HCC) tumorigenesis has also been described [7,9,10]. Indeed, on one hand, Cu can promote cell proliferation and activate oncogenic pathways; on the other hand, it may also counteract tumor growth by activating inflammatory cells, inducing oxidative stress, and regulating cell death pathways [7,11].

Zinc is essential for the structure and function of a large number of macromolecules, including over 300 enzymes [12]. An interesting aspect of Zn is its involvement in the structure of zinc finger proteins. As transcription factors, these proteins perform various fundamental regulatory functions within cells, and some of them appear to positively influence oncogenic pathways related to cell cycle control, replication, and the immune response [13,14].

This has prompted several authors to investigate the genetic signatures involved in Zn and Cu metabolism, particularly their association with HCC and inflammation. The findings increasingly support a role for the homeostasis of these metals in oncogenic pathways. These insights have led to the hypothesis that modulating metal homeostasis may offer therapeutic potential in patients with HCC [15,16,17].

Nonetheless, while evidence supports the involvement of these metals in hepatic tumorigenesis, the precise nature of this relationship and its underlying mechanisms remain unclear. Defining intracellular threshold levels above or below which harmful effects occur is particularly challenging, as these metals are essential for numerous cellular functions and overall biological balance.

In contrast to Zn and Cu, iron (Fe) presents a more complex picture. Patients with Fe storage disorders, such as hereditary hemochromatosis, exhibit an increased risk of HCC. It has also been hypothesized that Fe may exert an antitumor effect through its involvement in the activation of a cell death mechanism known as ferroptosis [18,19].

Circulating metal concentrations have also been proposed as possible biomarkers for diagnosing and predicting the prognosis of HCC. In particular, Zn has shown a protective role, whereas Cu has been associated with an increased risk of HCC and elevated transaminase and γGT activities [20,21,22,23,24]. Accordingly, an elevated serum Cu/Zn ratio in HCC patients has been identified as a significant prognostic marker associated with poorer outcomes and a higher risk of HCC progression [21,22,23,24,25].

Recent evidence also suggests greater avidity for Mg in cirrhotic patients with HCC, reflected in lower circulating serum concentrations of this metal. This may be due to its increased utilization in metabolic pathways essential for tumor proliferation [26].

To our knowledge, the tissue levels of Mg in HCC have not been investigated. However, experimental evidence has demonstrated both an upregulation of membrane transporters for this metal in tumor cells and a reduction in tumor growth following their inhibition, thus supporting the hypothesis of a potential role for Mg in tumorigenesis [27,28,29,30].

Further supporting the link between metals and tumorigenesis, several studies have analyzed the metal content in liver tissues in relation to HCC. Notably, higher Cu levels have been reported in tumor tissue compared to adjacent non-tumor or normal liver tissue. These studies, conducted primarily in Asian and Latino populations, often included patients without cirrhosis, and healthy liver samples were typically obtained post-mortem [1,31,32,33,34,35,36]. Additionally, the Cu content in cirrhotic tissue obtained during liver transplantation has been shown to be higher than in donor-derived normal liver tissue [20].

In contrast, the Zn content appears to be lower in HCC tissue than in non-tumor liver tissue [20,32,33,34,35,36,37]. An opposite trend to that of Cu has also been reported for the presence or absence of cirrhosis. Some, but not all, studies observed reduced Zn content in cirrhotic tissue compared to non-cirrhotic tissue [20,32,34,37].

The data regarding Fe are controversial, probably due to the heterogeneity between the cohorts analyzed in the different studies [20,31,33,34].

To date, no study has concurrently assessed the content of Cu, Zn, Fe, and Mg in healthy liver tissue, cirrhotic tissue from patients without HCC, cirrhotic tissue from patients with HCC, and HCC tissue itself, specifically within Western populations. Therefore, our study aims to be the first to compare the tissue content of these metals in different liver conditions and correlate them with serum markers of liver damage.

## 2. Results

### 2.1. Characteristics of the Study Population

**Figure 1:**
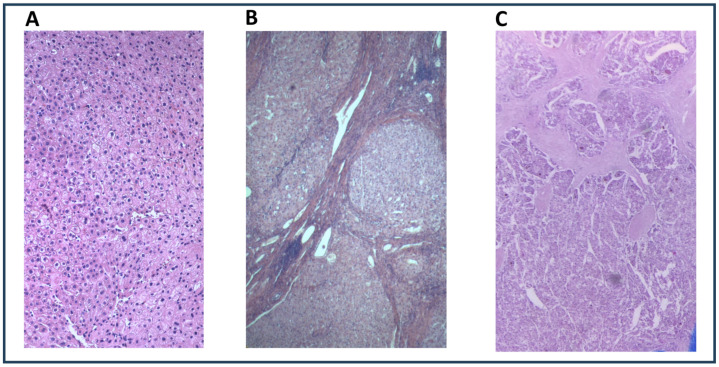
Histological examples of a healthy donor liver at original magnification 10× (panel (**A**)), cirrhotic liver tissue at original magnification 2× (panel (**B**)), and HCC tissue at original magnification 2× (panel (**C**)).

Figure 1 shows histological examples of a healthy donor liver, cirrhotic liver tissue, and HCC tissue. All HCCs were grade G2.

Table 1 shows the demographic and clinical characteristics of liver transplant donors with healthy livers, cirrhotic patients without HCC, and cirrhotic patients with HCC. There were no differences in age, gender, and BMI between the groups. Donors had significantly lower serum aminotransferase activity than the two groups of patients with cirrhosis, which did not differ from each other. Cirrhotic patients with HCC had significantly higher Model for End stage Liver Disease (MELD) scores and γGT activities than patients with cirrhosis only.

### 2.2. Metal Content in Liver Tissue

Firstly, we investigated the metal content of liver tissues.

**Figure 2:**
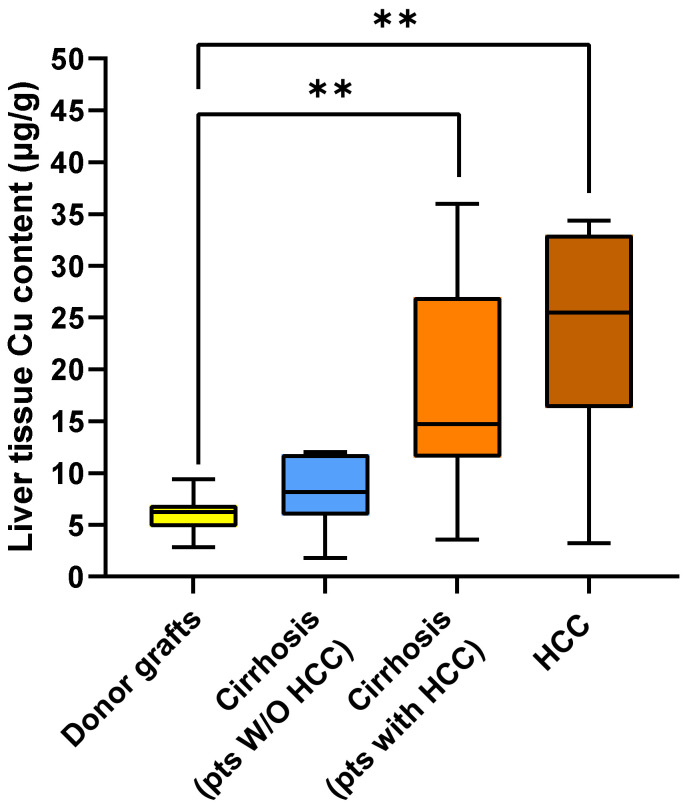
Box plot of copper content measured in the livers of donors, in the cirrhotic tissue of patients without and with HCC, and in tumor tissue. Significant *p* values are reported as follows: ** = *p* < 0.01.

In this regard, Figure 2 shows the Cu content in the four liver tissue groups analyzed in the study. Interestingly, the median Cu content was increased progressively from the livers of transplant donors [6.26 (IQR 4.88–6.85) µg/g], to cirrhotic tissue from patients without HCC [8.15 (IQR 6.39–11.75) µg/g], to cirrhotic tissue from patients with HCC [14.73 (IQR 12.28–24.82) µg/g], and to HCC tissue from patients with HCC [25.50 (IQR 17.00–32.59) µg/g] (*p* for trend = 0.000004).

Comparing the Cu content in different tissues, we observed that the Cu content was significantly lower in donor livers compared to both cirrhotic liver from HCC patients (*p* < 0.01) and tumor tissue (*p* < 0.01). However, no significant differences in tissue Cu content were found between the livers of donors and the cirrhotic livers of patients without HCC, nor when comparing the three diseased liver groups.

**Figure 3:**
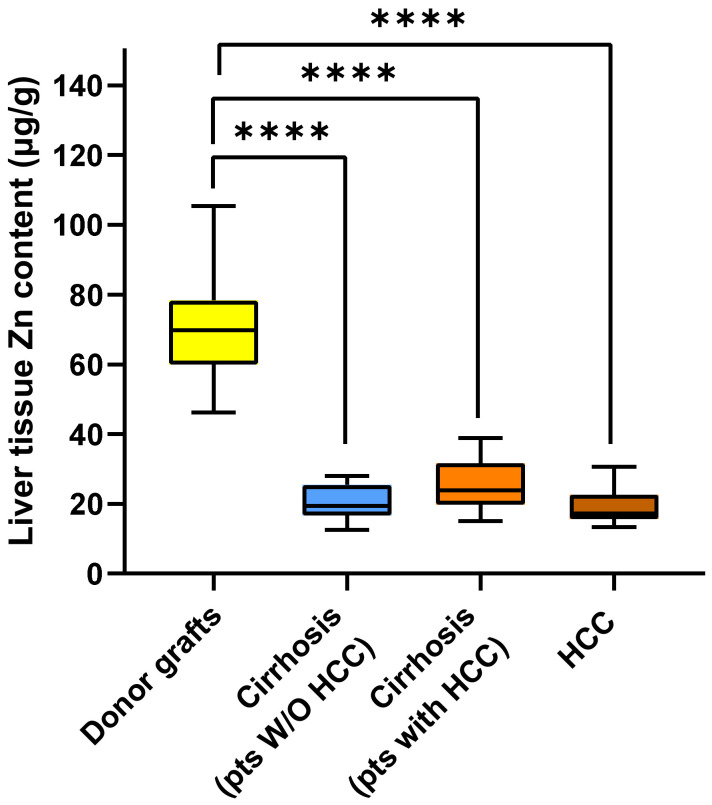
Box plot of zinc content measured in the liver of donors, in the cirrhotic tissue of patients without and with HCC, and in tumor tissue. Significant *p* values are reported as follows: **** = *p* < 0.0001.

Figure 3 shows the median Zn content in the tissues. Unlike Cu, the median Zn content was significantly higher in liver transplant donors [69.79 (IQR 60.69–77.61) µg/g] than in cirrhotic tissue from patients without HCC [19.47 (IQR 16.81–25.18) µg/g; *p* < 0.00001], in cirrhotic tissue from patients with HCC [23.86 (IQR 20.12–31.05) µg/g; *p* < 0.0001], and in HCC tissue from patients with HCC [17.26 (IQR 15.87–20.47) µg/g; *p* < 0.0001]. However, the three groups with diseased livers did not differ significantly from each other with regard to tissue Zn content.

**Figure 4:**
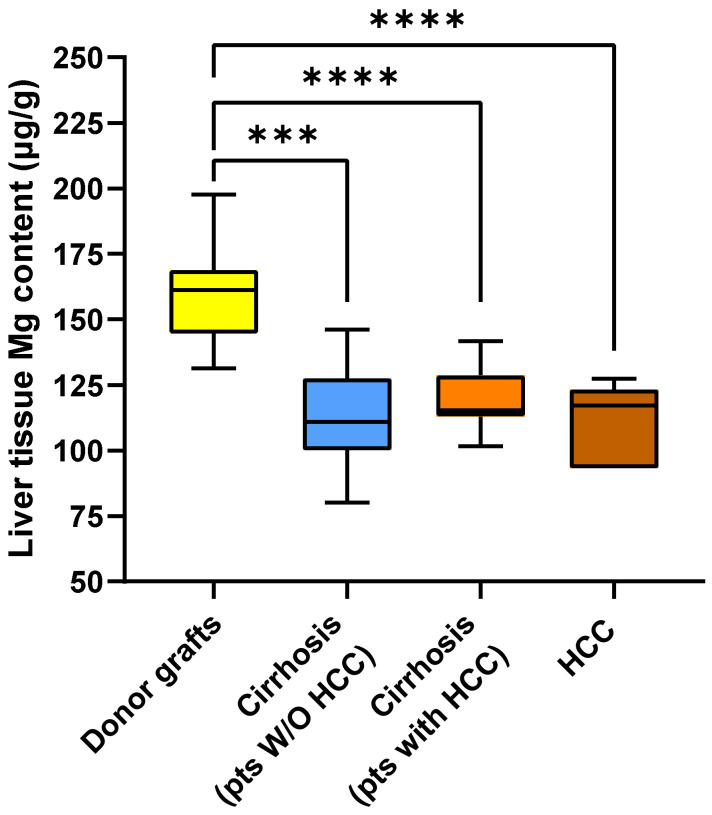
Box plot of magnesium content measured in the liver of donors, in the cirrhotic tissue of patients without and with HCC, and in tumor tissue. Significant *p* values are reported as follows: *** = *p* < 0.001; **** = *p* < 0.0001.

As shown in Figure 4, the median Mg content was significantly higher in liver transplant donors [161.29 (IQR 144.64–168.33) µg/g] than in cirrhotic tissue from patients without HCC [110.91 (IQR 101.20–125.66) µg/g; *p* < 0.001], in cirrhotic tissue from patients with HCC [115.27 (IQR 113.13–125.90) µg/g; *p* < 0.0001], and in HCC tissue from patients with HCC [117.15 (IQR 109.35–122.5) µg/g; *p* < 0.0001]. The three groups with diseased livers did not differ significantly from each other with regard to tissue Mg content.

**Figure 5:**
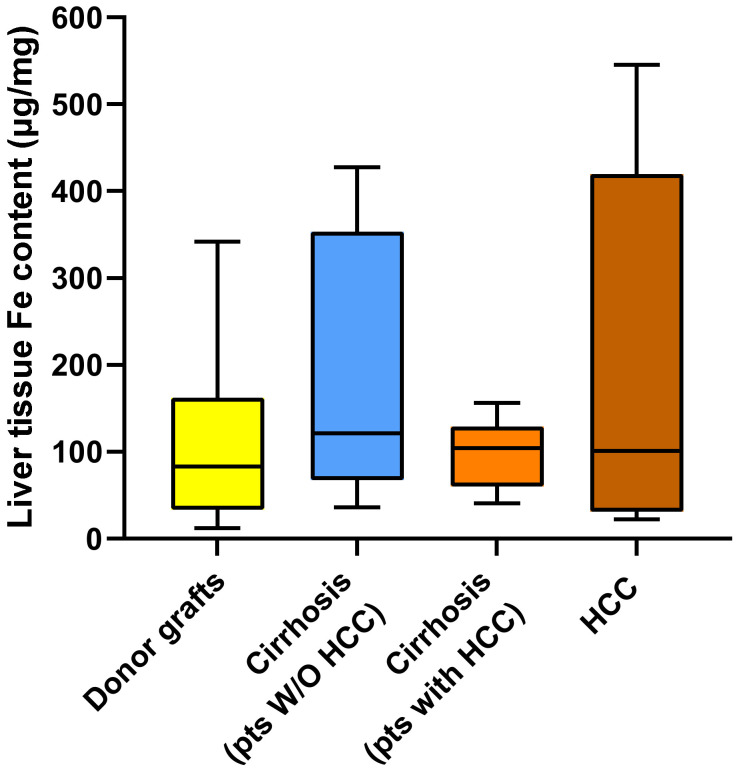
Box plot of iron content measured in the livers of donors, in the cirrhotic tissue of patients without and with HCC, and in tumor tissue.

The median Fe content did not differ between any of the groups, with values of [83.19 (IQR 36.0–156.98) µg/g], [121.29 (IQR 69.91–350.74) µg/g], [104.23 (IQR 60.25–120.12) µg/g], and [100.93 (IQR 31.76–391.04) µg/g] in the livers of donors, in the cirrhotic tissue of patients without HCC, in the cirrhotic tissue of patients with HCC, and in HCC tissue, respectively (Figure 5).

**Figure 6:**
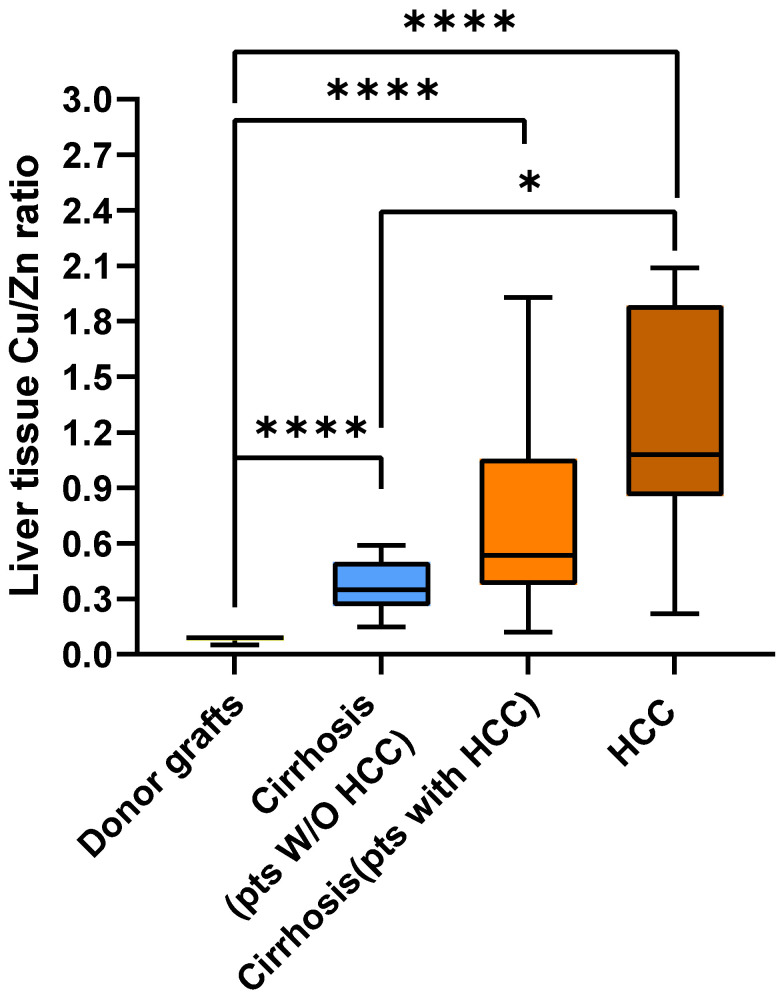
Box plot of copper/zinc ratio in the livers of donors, in the cirrhotic tissue of patients without and with HCC, and in tumor tissue. Significant *p* values are reported as follows: * = *p* < 0.05, **** = *p* < 0.0001.

We then aimed to investigate whether the Cu/Zn ratio differed among the four liver tissue groups. As shown in Figure 6, the median Cu/Zn ratio increased progressively from liver transplant donors [0.091 (IQR 0.076–0.098)], to cirrhotic tissue from patients without HCC [0.350 (IQR 0.270–0.467)], to cirrhotic tissue from patients with HCC [0.535 (IQR 0.376–1.026)], and to HCC tissue from patients with HCC [1.080 (IQR 0.872–1.823)] (*p* for trend = 1.9955 × 10^−10^). The Cu/Zn ratio was significantly lower in donor livers than in each of the three diseased liver groups (*p* < 0.0001). Furthermore, the Cu/Zn ratio was significantly lower in cirrhotic tissue from patients without HCC than in HCC tissue (*p* < 0.05).

### 2.3. Correlations Between Tissue Metal Content and Serum ALT and γGT

Finally, we investigated whether there were any correlations between the tissue Cu content and serum ALT and γGT activity analyzed separately within donors, cirrhotic patients without HCC, and patients with HCC. As shown in Table 2, a positive correlation was found between tissue Cu content and serum ALT activity in donors. In cirrhotic patients without HCC, tissue Cu content was positively correlated with serum ALT and γGT activity. However, these correlations were absent in cirrhotic tissue from patients with HCC.

## 3. Discussion

In this study, we investigated the association between Cu, Zn, Mg, Fe and hepatic tissues from different liver diseases. Specifically, we examined normal liver tissue from liver transplantation donors, cirrhotic liver tissue from patients without HCC, cirrhotic liver tissue from patients with HCC, and HCC tissue from the same patients in the latter group. This comprehensive approach allowed us to identify significant differences in metal content across these tissue types.

One of the most interesting findings of our study was the significant increase in Cu content in cirrhotic tissue obtained from HCC patients and in tumor tissue compared to normal liver, while cirrhotic tissue from patients without HCC did not differ from normal liver tissue. Notably, the median Cu content in tumor tissue and cirrhotic tissue from these patients was four and two times higher, respectively, than in healthy liver tissue.

These results are consistent with previous studies suggesting a high tissue Cu content in HCC, with the added value of also offering, in Western subjects, new simultaneous comparative data between cirrhotic tissue from patients with and without HCC [11,31,32,33,34,35,36].

Although observational in nature, our data could be relevant to the pathogenesis of HCC. Indeed, we found that both HCC and cirrhotic tissue from HCC patients contained more Cu than healthy livers but not cirrhotic tissue from non-HCC patients. While our study does not provide data on this, it is likely that the mechanism of Cu enrichment in HCC tissue and in cirrhotic tissue of patients with this type of tumor is due to its excessive cellular uptake. This hypothesis is supported by recent experimental data on the malignancy-promoting role of the Six Transmembrane Epithelial Antigen of Prostate 2 (STEAP2) in HCC cells, which indirectly contributes to the cellular entry of Cu [11]. However, the oncogenic mechanism of Cu is unclear for two reasons. Firstly, although a higher severity of cirrhosis is considered a risk factor for the development of HCC, in our study, a high Cu content in both tumor and cirrhotic tissue was associated with a lower cirrhosis severity, as indicated by lower MELD score values [38,39,40,41,42]. Secondly, although high γGT activity have previously been associated with an increased risk of HCC and in our patients with HCC it was higher than in cirrhotic patients without HCC, the tissue Cu content correlated with γGT in cirrhotic patients without HCC but not in those with HCC [43,44,45]. Therefore, although our study does not provide causal data, it is possible that the high Cu content in tissues is an oncogenic factor unrelated to the severity of cirrhosis, the extent of oxidative stress or the degree of liver inflammation related to the severity of cirrhosis, as represented by γGT activity [6,9,10,11,46]. As demonstrated in other studies, excessive Cu^+^ accumulation in cells could lead, per se, to the production of reactive oxygen species and cellular damage, increasing oxidative stress, inflammation, and DNA damage, with subsequent stimulation of cell proliferation and angiogenesis [47]. Although we did not investigate this aspect in our study, there is also emerging research on the oncogenic role of cuproptosis, a novel form of copper-induced cell death mediated by protein lipoylation [47]. The interplay between HCC and cuproptosis represents a nascent but rapidly evolving area of investigation, focusing on the regulatory role of copper metabolism and the contribution of cuproptosis in HCC initiation and progression [48].

In contrast to Cu, normal liver tissue exhibited a significantly higher Zn content than all of the diseased liver tissues analyzed, and there were no differences between the diseased liver groups. Our in vivo data confirm the previous finding of a lower Zn content in HCC tissue compared to normal liver obtained at autopsy [32,33,35,37]. Regarding the comparison between HCC and cirrhotic liver tissue, our data do not show significant differences, even though cirrhosis in HCC patients had slightly higher values than HCC tissue. These data are in disagreement with previous studies where non-tumor tissue showed significantly higher values [33,34,35,36,37]. These differences may be due to a lower severity of chronic liver disease in these studies compared to our patients, who were candidates for liver transplantation. In fact, in most of these studies, many or all patients did not have cirrhosis [33,34,35,36]. In the one study (including only black cirrhotic patients) where tissue sampling was performed at autopsy, no data are reported for the severity of cirrhosis, which may have been lower than in our patients [37].

The data from our study do not allow us to hypothesize the causes of the reduced Zn content in diseased liver tissue compared to controls. In patients with advanced chronic liver disease, several possible mechanisms have been described, including alterations in hepatocyte cellular transport mediated by inflammatory cytokines, redistribution in other body compartments secondary to the opening of spontaneous portosystemic shunts, and intestinal malabsorption and increased urinary loss [32,33,35,37,49]. Therefore, our data indirectly support the possibility that Zn plays an overall protective role against hepatic oncogenesis and that its reduction may favor HCC development. Indeed, even with regard to the possible oncogenic role of Zn reduction in liver cells, our study does not provide causal data. We can only speculate, as demonstrated in other studies, that Zn deficiency may promote the cell cycle and reduce apoptosis, the latter through the reduction of metallothienin synthesis, oxidative stress, and a reduction in the ability to repair DNA [47]. Finally, a Zn reduction in the tumor microenvironment could also inhibit the normal immunological response against tumor cells [47].

For the first time, we also evaluated the Cu/Zn ratio (which has previously only been studied in blood) in different types of liver tissue [21,22,23,24,25]. We confirmed the association between a high Cu/Zn ratio and HCC at the tissue level. We observed a progressive increase in Cu/Zn ratio at the tissue level from healthy liver tissue to cirrhotic liver without HCC, to cirrhotic liver with HCC, and finally to HCC tissue. Furthermore, pairwise comparisons demonstrated significant differences between healthy liver tissues and all other tissues and showed that the values in cirrhotic tissue from patients without HCC were significantly lower than those in HCC tissue. Therefore, the higher Cu/Zn ratio at the tissue level could favor tumorigenic pathways. However, this remains a speculative interpretation, since the mechanisms underlying this observation cannot be definitively established. Although this aspect was also not investigated in our study, the inverse interplay between Cu and Zn could be mediated by metallothioneins, a family of small, cysteine-rich, ubiquitous proteins that bind various metals, including Cu and Zn. Kubo et al. (2005) found that normal hepatic tissue showed an absence of Cu bound to metallothionein, whereas the Cu content bound to this protein in tumors was significantly higher than that in a normal liver [32]. Conversely, the content of Zn bound to metallothionein in tumors was significantly lower than that in a normal liver.

Similar to Zn, Mg showed comparable content in cirrhotic liver tissue from patients with and without HCC, as well as in HCC tissue, but a higher content was observed in healthy liver tissue compared to diseased liver. This is in line with the previous results of our group comparing cirrhotic and normal liver tissue [50]. However, in our current study, we did not find higher values in HCC tissue than in cirrhotic tissue from the same patients. These results do not support our previous hypothesis, which was based exclusively on serological data and which indirectly suggested that the tumor exhibits high Mg avidity [26].

In contrast to other metals investigated, our study did not reveal any significant differences in tissue Fe content. This finding contributes to the overall heterogeneity of existing data on Fe levels in cirrhotic livers and HCC [31,33,34]. One possible explanation for this variability is that the potentially deleterious/oncogenic versus tumor-suppressive roles of Fe may differ substantially depending on the specific conditions, tissue content, and underlying hepatic pathology. However, a limitation of our study is that we did not measure the blood concentrations of metals. In addition to correlating the tissue Cu/Zn ratio with the serum ratio, it would have been important to correct the tissue Fe data for circulating Fe, also considering that Fe is bound to hemoglobin, which can be influenced by a history of anemia, red blood cell transfusions, and iron therapy. Therefore, studies involving larger patient cohorts and correcting for circulating Fe values are needed to verify whether elevated tissue Fe levels may or may not be associated with HCC. As for Cu, Zn, and Mg, it is unlikely that circulating amounts of these metals could have altered the results. Indeed, the extracellular concentrations of Cu, Zn, and Mg are approximately 300, 100, and 100 times lower than their concentration in cells, respectively [51,52]. 

Our study has additional limitations that must be acknowledged. First, it is a single-center early exploratory study with mainly descriptive results. Indeed, because the literature on the subject is inconsistent and scarce, we did not perform a formal sample size calculation. This resulted in relatively small numbers for each group that did not allow for in-depth multivariate statistical analyses, thus significantly reducing the ability to support causality and the independence of our results. Second, it was not possible to evaluate the metal content in relation to the histopathological features of liver tissues and HCC. Furthermore, by excluding the four HCC tissues with locoregional treatment-induced necrosis, a selection bias may have been introduced by excluding the most aggressive tumors. If this were true, it is likely that the differences between the HCC group and the other groups were diminished by these exclusions. As a final limitation of our study, we did not analyze potential confounders such as some complications of cirrhosis, drug therapies, nutritional status, or ceruloplasmin and metallothionein levels. Nevertheless, a major strength of our study is its comprehensive evaluation of key metals involved in fundamental cellular processes across different types of hepatic tissue, providing new data that have not been documented in previous studies. In fact, although our study is descriptive, it has several strengths compared to previous studies that analyzed metal content in HCC [11,20,31,32,33,34,35,36]. First, we analyzed the hepatic content of four metals in HCC and cirrhotic liver samples from the same patients, which were taken at an adequate distance from the tumor and not in the tissue surrounding the tumor. Second, our study also compared these tissues with the cirrhotic livers of patients without HCC and with healthy livers, not using any samples obtained at autopsy. Finally, with the sole exception of the few cases enrolled in a previous study in which only hepatic Cu was measured, our study is the only one that enrolled Caucasian subjects [20].

Based on our findings, we believe that further research is essential to better elucidate the relationship between tissue metal content and HCC oncogenesis. If these associations are confirmed, it may be worthwhile to investigate the potential therapeutic implications of pharmacological agents targeting metal homeostasis in this malignancy through experimental studies.

## 4. Materials and Methods

### 4.1. Study Design

The present study is a single-center retrospective study that was conducted at Sapienza University of Rome, Azienda Ospedaliero-Universitaria Policlinico Umberto I, Rome, Italy.

In this study, liver tissue samples and clinical data were analyzed from cirrhotic patients undergoing liver transplantation and from liver donors. The study was conducted in accordance with the Declaration of Helsinki and was approved by the Ethics Committee of Sapienza University-Policlinico Umberto I (Ref. N. 3420/27/11/2014).

### 4.2. Study Population and Data Collection

A total of 14 cirrhotic patients with HCC, 14 cirrhotic patients without HCC, and 18 liver donors were enrolled in this study.

Liver tissue samples were collected from each subject enrolled in the study at the time of liver transplantation in cirrhotic patients or at the time of organ procurement in deceased donors. All liver tissue samples were analyzed to determine their metal content.

The following exclusion criteria were applied for both donors and cirrhotic patients: age > 70 years and acute or chronic renal failure with an estimated glomerular filtration rate < 50. Donors with any degree of hepatic steatosis were excluded. In addition, for cirrhotic patients, treatment with any supplement containing Mg, Zn, Cu, vitamin D, calcium, or bisphosphonates in the 6 months prior to enrollment, acute alcoholic hepatitis or acute-on-chronic liver failure, Wilson’s disease, hemochromatosis, and thyroid disease were excluded.

Liver tissue samples were obtained through wedge biopsies performed on the left hepatic lobe immediately after liver explantation in cirrhotic livers and before ischemia in donors. In cirrhotic patients with HCC, two wedge biopsies were performed, one on the tumor tissue and one on cirrhotic tissue at least 5 cm from the tumor. Half of each biopsy was immediately fixed in 10% neutral buffered formalin and then sections were created in paraffin and stained with hematoxylin and eosin for histological confirmation of tissue type. The other half was rapidly frozen and stored in liquid nitrogen until metal analysis. In 4 of the cirrhotic patients with HCC, the tumor tissue was largely necrotic due to locoregional treatments performed as a bridge to transplantation and, therefore, was not analyzed for metal content. Thus, according to the sampling site, the analyzed liver tissues were donor graft (*n* = 18), cirrhotic tissue from patients without HCC (*n* = 14), cirrhotic tissue from patients with HCC (*n* = 14), HCC tissue (*n =* 10). Clinical data were collected in the hours preceding surgery.

### 4.3. Study Aims

The primary aim of this study was to evaluate whether the content of metals (Zn, Cu, Fe, and Mg) in liver tissue varies according to the presence or absence of liver cirrhosis and HCC. As a secondary objective, we also investigated the possible correlations between the metal content in normal livers and the cirrhotic tissues in patients with and without HCC and ALT and γGT activities, which are serum markers of liver and cholangiocyte damage, respectively.

### 4.4. Metal Quantification in Liver Tissue Using Atomic Absorption Spectrometry

Liver tissue sample preparation and analysis were carried out according to a previously published protocol [50]. Biopsies of liver tissue were placed in individual acid-washed Teflon jars and were digested with 2 mL 65% HNO_3_ and 0.5 mL 30% H_2_O_2_ in a microwave oven for 5 min at 250 W, 5 min at 400 W, 5 min at 500 W, and finally 1 min at 600 W. The cooled samples were transferred to polyethylene volumetric flasks, diluted to 10 mL, and analyzed using a flame atomic spectrophotometer (AAnalyst 100, Perkin Elmer, Waltham, MA, USA). Iron, Zn, and Cu were analyzed by direct aspiration of the solutions into the flame of the spectrophotometer, while Mg was analyzed after diluting the solutions 1:10. The accuracy of the method was evaluated using the analysis of an internationally certified reference material (ERM^®^-BB422 fish muscle). The metal content determined with the method used in this study fell within the certified uncertainty interval provided by ERM, corresponding to a 95% confidence level. The detection limits for flame atomic absorption spectroscopy were 0.04 µg/mL for Mg, 0.09 µg/mL for Fe, 0.04 µg/mL for Zn, and 0.01 µg/mL for Cu. The metal content in liver biopsies was expressed as µg/g wet weight (ww). All the reagents were purchased from Merck (Merck-Sigma-Aldrich, Darmstadt, Germany) and were of Suprapur grade.

### 4.5. Statistical Methods

There were no missing data in the study, therefore no imputations were performed. Continuous variables were assessed for normality using the Shapiro–Wilk test and subsequently expressed as either the median and interquartile range (IQR) or mean and standard deviation (SD), depending on their distribution. Categorical data are presented as frequencies and percentages.

Differences in demographic and clinical characteristics between donors and patients with cirrhosis with and without HCC and within the latter two groups were assessed using the Mann–Whitney U test, *t*-test, or chi-square test as appropriate.

Differences in the metal content among the four tissue types were first analyzed with the Jonckheere–Terpstra test for ordered alternatives in k samples and then by pairwise multiple comparisons with Bonferroni correction.

Correlations between the tissue metal content and serum ALT and GGT activities were assessed using Pearson’s r coefficient.

All tests were two-tailed, and a *p*-value < 0.05 was considered statistically significant. Statistical analyses were performed using SPSS 27.0 (SPSS Inc., Chicago, IL, USA).

## Figures and Tables

**Table 1:** Demographic and clinical characteristics of the study population.

	Liver Graft Donors (n = 18)	Cirrhotic Patients Without HCC (n = 14)	Cirrhotic Patients with HCC (n = 14)	*p* Value Donors vs. Cirrhotic Patients Without HCC	*p* Value Donors vs. Cirrhotic Patients with HCC	*p* Value Cirrhotic Patients Without HCC vs. Those with HCC
Age (years)	53.00 (37.00–62.00)	52.00 (47.25–56.00)	53.50 (48.00–63.75)	0.892	0.296	0.168
Sex M (%)	12 (66.7)	10 (71.4)	13 (92.9)	1.000	0.104	0.326
BMI (kg/m^2^)	25.70 (24.95–27.31)	24.20 (22.89–27.42)	24.85 (22.65–29.98)	0.184	0.569	0.818
Serum AST (IU/L)	33.50 (22.00–43.75)	58.50 (41.75–92.00)	75.50 (39.75–117.25)	0.006	0.006	0.520
Serum ALT (IU/L)	23.00 (18.00–29.50)	48.00 (25.00–59.25)	51.00 (36.75–117.00)	0.008	0.001	0.232
Serum ALP (IU/L)	Not available	137.00 (105.00–176.50)	110.00 (83.50–204.25)	_	_	0.713
Serum γGT (IU/L)	Not available	40.00 (29.75–50.00)	104.00 (58.25–129.50)	_	_	0.0004
MELD score	Not applicable	19.06 (12.70–22.83)	10.36 (7.75–14.12)	_	_	0.008
Etiology of Liver Cirrhosis, n (%)						
Viral	Not applicable	8 (57.1)	12 (85.7)	_	_	0.103
Alcohol		6 (42.9)	5 (35.7)	_	_	1.000
MASLD		2 (14.3)	4 (28.6)	_	_	0.648

Abbreviations: ALT, alanine aminotransferase; ALP, alkaline phosphatase; AST, aspartate aminotransferase; BMI, body mass index; γGT, gamma-glutamyl transferase; HCC, hepatocellular carcinoma; MASLD, metabolic associated steatotic liver disease; MELD, Model for End stage Liver Disease.

**Table 2:** Correlations between tissue Cu content and serum ALT and γGT activity.

Tissue Cu Content	Serum ALT		Serum γGT	
	r	*p* Value	r	*p* Value
Donor grafts	0.520	0.027	N.A.	N.A.
Cirrhotic tissue (pts W/O HCC)	0.873	<0.001	0.846	<0.001
Cirrhotic tissue (pts with HCC)	−0.006	0.983	−0.004	0.990

Abbreviations: ALT, alanine aminotransferase; Cu, copper; γGT, gamma-glutamyltransferase; HCC, hepatocellular carcinoma; W/O, without; Zn, zinc.

## Data Availability

The data in this study are available from the corresponding authors upon reasonable request.

## Abbreviations

The following abbreviations are used in this manuscript:

ALT	Alanine aminotransferase
BMI	Body mass index
Cu	Copper
Fe	Iron
IQR	Interquartile range
HCC	Hepatocellular carcinoma
LT	Liver transplantation
MASLD	Metabolic associated steatotic liver disease
MELD	Model for End stage Liver Disease
Mg	Magnesium
Pts	Patients
Zn	Zinc
W/O	Without

## References

[1] Mendes P.M.V., Bezerra D.L.C., dos Santos L.R., Santos R.d.O., Melo S.R.d.S., Morais J.B.S., Severo J.S., Vieira S.C., Marreiro D.D.N. (2018). Magnesium in Breast Cancer: What Is Its Influence on the Progression of This Disease?. Biol. Trace Elem. Res..

[2] Torti S.V., Manz D.H., Paul B.T., Blanchette-Farra N., Torti F.M. (2018). Iron and Cancer. Annu. Rev. Nutr..

[3] Ho E., Song Y. (2009). Zinc and prostatic cancer. Curr. Opin. Clin. Nutr. Metab. Care.

[4] Bao Y., Zhang H., Han Z., Guo Y., Yang W. (2022). Zinc Fingers and Homeobox Family in Cancer: A Double-Edged Sword. Int. J. Mol. Sci..

[5] Guo J., Cheng J., Zheng N., Zhang X., Dai X., Zhang L., Hu C., Wu X., Jiang Q., Wu D. (2021). Copper Promotes Tumorigenesis by Activating the PDK1-AKT Oncogenic Pathway in a Copper Transporter 1 Dependent Manner. Adv. Sci..

[6] Lin J., Luo B., Yu X., Yang Z., Wang M., Cai W. (2022). Copper metabolism patterns and tumor microenvironment characterization in colon adenocarcinoma. Front. Oncol..

[7] Kamiya T. (2022). Copper in the tumor microenvironment and tumor metastasis. J. Clin. Biochem. Nutr..

[8] Linder M.C., Hazegh-Azam M. (1996). Copper biochemistry and molecular biology. Am. J. Clin. Nutr..

[9] Wu Z., Lv G., Xing F., Xiang W., Ma Y., Feng Q., Yang W., Wang H. (2023). Copper in hepatocellular carcinoma: A double-edged sword with therapeutic potentials. Cancer Lett..

[10] Zhou C., Yang J., Liu T., Jia R., Yang L., Sun P., Zhao W. (2023). Copper metabolism and hepatocellular carcinoma: Current insights. Front. Oncol..

[11] Torrez C.Z., Easley A., Bouamar H., Zheng G., Gu X., Yang J., Chiu Y.-C., Chen Y., Halff G.A., Cigarroa F.G. (2024). STEAP2 promotes hepatocellular carcinoma progression via increased copper levels and stress-activated MAP kinase activity. Sci. Rep..

[12] Tapiero H., Tew K.D. (2003). Trace elements in human physiology and pathology: Zinc and metallothioneins. Biomed. Pharmacother..

[13] Li X., Han M., Zhang H., Liu F., Pan Y., Zhu J., Liao Z., Chen X., Zhang B. (2022). Structures and biological functions of zinc finger proteins and their roles in hepatocellular carcinoma. Biomark. Res..

[14] Cassandri M., Smirnov A., Novelli F., Pitolli C., Agostini M., Malewicz M., Melino G., Raschellà G. (2017). Zinc-finger proteins in health and disease. Cell Death Discov..

[15] Shi H., Huang J., Wang X., Li R., Shen Y., Jiang B., Ran J., Cai R., Guo F., Wang Y. (2023). Development and validation of a copper-related gene prognostic signature in hepatocellular carcinoma. Front. Cell Dev. Biol..

[16] Kong L., Liu M., Yang H., Yan P., Luo Y., Xiang S., Huang Z., Shen A. (2024). Expression of copper metabolism-related genes is associated with the tumor immune microenvironment and predicts the prognosis of hepatocellular carcinoma. Transl. Cancer Res..

[17] Shi Y., Ye R., Gao Y., Xia F., Yu X.F. (2024). A prognostic and immune related risk model based on zinc homeostasis in hepatocellular carcinoma. iScience.

[18] Li X., Meng F., Wang H., Sun L., Chang S., Li G., Chen F. (2024). Iron accumulation and lipid peroxidation: Implication of ferroptosis in hepatocellular carcinoma. Front. Endocrinol..

[19] Kowdley K.V. (2004). Iron, hemochromatosis, and hepatocellular carcinoma. Gastroenterology.

[20] Poznański J., Sołdacki D., Czarkowska-Pączek B., Bonna A., Kornasiewicz O., Krawczyk M., Bal W., Pączek L. (2021). Cirrhotic Liver of Liver Transplant Recipients Accumulate Silver and Co-Accumulate Copper. Int. J. Mol. Sci..

[21] Stepien M., Hughes D.J., Hybsier S., Bamia C., Tjønneland A., Overvad K., Affret A., His M., Boutron-Ruault M.-C., Katzke V. (2017). Circulating copper and zinc levels and risk of hepatobiliary cancers in Europeans. Br. J. Cancer.

[22] Liu X., Zhang Y., Yishake D., Luo Y., Liu Z., Chen Y., Zhu H., Fang A. (2024). Dietary intake and serum levels of copper and zinc and risk of hepatocellular carcinoma: A matched case-control study. Chin. Med. J..

[23] Tamai Y., Iwasa M., Eguchi A., Shigefuku R., Sugimoto K., Hasegawa H., Takei Y., Alpini G.D. (2020). Serum copper, zinc and metallothionein serve as potential biomarkers for hepatocellular carcinoma. PLoS ONE.

[24] Poo J.L., Rosas-Romero R., Montemayor A.C., Isoard F., Uribe M. (2003). Diagnostic value of the copper/zinc ratio in hepatocellular carcinoma: A case control study. J. Gastroenterol..

[25] Fang A., Chen P., Wang X., Liu Z., Zhang D., Luo Y., Liao G., Long J., Zhong R., Zhou Z. (2019). Serum copper and zinc levels at diagnosis and hepatocellular carcinoma survival in the Guangdong Liver Cancer Cohort. Int. J. Cancer.

[26] Parisse S., Ferri F., Persichetti M., Mischitelli M., Abbatecola A., Di Martino M., Lai Q., Carnevale S., Lucatelli P., Bezzi M. (2021). Low serum magnesium concentration is associated with the presence of viable hepatocellular carcinoma tissue in cirrhotic patients. Sci. Rep..

[27] Voringer S., Schreyer L., Nadolni W., Meier M.A., Woerther K., Mittermeier C., Ferioli S., Singer S., Holzer K., Zierler S. (2020). Inhibition of TRPM7 blocks MRTF/SRF-dependent transcriptional and tumorigenic activity. Oncogene.

[28] Yu Z., Song Y., Cai M., Jiang B., Zhang Z., Wang L., Jiang Y., Zou L., Liu X., Yu N. (2021). PPM1D is a potential prognostic biomarker and correlates with immune cell infiltration in hepatocellular carcinoma. Aging.

[29] Li Q., Xiong D.L., Wang H., Jin W.L., Ma Y.Y., Fan X.M. (2021). High Expression of SLC41A3 Correlates with Poor Prognosis in Hepatocellular Carcinoma. Onco Targets Ther..

[30] Flannery P.C., Abbott K.L., Pondugula S.R. (2020). Correlation of PPM1A Downregulation with CYP3A4 Repression in the Tumor Liver Tissue of Hepatocellular Carcinoma Patients. Eur. J. Drug Metab. Pharmacokinet..

[31] Geetha A., Saranya P., Annie Jeyachristy S., Surendran R., Sundaram A. (2009). Relevance of Non-ceruloplasmin Copper to Oxidative Stress in Patients with Hepatocellular Carcinoma. Biol. Trace Elem. Res..

[32] Kubo S., Fukuda H., Ebara M., Ikota N., Saisho H., Nakagawa H., Ozawa T., Yukawa M., Kato K., Satoh T. (2005). Evaluation of Distribution Patterns for Copper and Zinc in Metallothionein and Superoxide Dismutase in Chronic Liver Diseases and Hepatocellular Carcinoma Using High-Performance Liquid Chromatography (HPLC). Biol. Pharm. Bull..

[33] Ebara M., Fukuda H., Hatano R., Yoshikawa M., Sugiura N., Saisho H., Kondo F., Yukawa M. (2003). Metal Contents in the Liver of Patients with Chronic Liver Disease Caused by Hepatitis C Virus. Oncology.

[34] Tashiro H., Kawamoto T., Okubo T., Koide O. (2003). Variation in the Distribution of Trace Elements in Hepatoma. Biol. Trace Elem. Res..

[35] Tashiro-Itoh T., Ichida T., Matsuda Y., Satoh T., Sugiyama M., Tanaka Y., Ishikawa T., Itoh S., Nomoto M., Asakura H. (1997). Metallothionein expression and concentrations of copper and zinc are associated with tumor differentiation in hepatocellular carcinoma. Liver.

[36] Ebara M., Fukuda H., Hatano R., Saisho H., Nagato Y., Suzuki K., Nakajima K., Yukawa M., Kondo F., Nakayama A. (2000). Relationship between copper, zinc and metallothionein in hepatocellular carcinoma and its surrounding liver parenchyma. J. Hepatol..

[37] Kew M.C., Mallett R.C. (1974). Hepatic zinc concentrations in primary cancer of the liver. Br. J. Cancer.

[38] Angelico M., Cillo U., Fagiuoli S., Gasbarrini A., Gavrila C., Marianelli T., Costa A.N., Nardi A., Strazzabosco M., Burra P. (2011). Liver Match, a prospective observational cohort study on liver transplantation in Italy: Study design and current practice of donor–recipient matching. Dig. Liver Dis..

[39] Fattovich G., Stroffolini T., Zagni I., Donato F. (2004). Hepatocellular carcinoma in cirrhosis: Incidence and risk factors. Gastroenterology.

[40] Goldberg D., French B., Newcomb C., Liu Q., Sahota G., Wallace A.E., Forde K.A., Lewis J.D., Halpern S.D. (2016). Patients with Hepatocellular Carcinoma Have Highest Rates of Wait-listing for Liver Transplantation Among Patients with End-Stage Liver Disease. Clin. Gastroenterol. Hepatol..

[41] Samuel D., De Martin E., Berg T., Berenguer M., Burra P., Fondevila C., Heimbach J.K., Pageaux G.-P., Sanchez-Fueyo A., Toso C. (2024). EASL Clinical Practice Guidelines on liver transplantation. J. Hepatol..

[42] Samuel D., Coilly A. (2018). Management of patients with liver diseases on the waiting list for transplantation: A major impact to the success of liver transplantation. BMC Med..

[43] Carr B.I., Akkiz H., Bag H.G., Karaoğullarından U., Yalçın K., Ekin N., Özakyol A., Altıntaş E., Balaban H.Y., Şimşek H. (2021). Serum levels of gamma-glutamyl transpeptidase in relation to HCC human biology and prognosis. J. Transl. Sci..

[44] Hu G., Tuomilehto J., Pukkala E., Hakulinen T., Antikainen R., Vartiainen E., Jousilahti P. (2008). Joint effects of coffee consumption and serum gamma-glutamyltransferase on the risk of liver cancer†. Hepatology.

[45] Sun P., Li Y., Chang L., Tian X. (2019). Prognostic and clinicopathological significance of Gamma-Glutamyltransferase in patients with hepatocellular carcinoma. Medicine.

[46] Lee D.H., Blomhoff R., Jacobs D.R. (2004). ReviewIs Serum Gamma Glutamyltransferase a Marker of Oxidative Stress?. Free Radic Res..

[47] Himoto T., Masaki T. (2024). Current Trends on the Involvement of Zinc, Copper, and Selenium in the Process of Hepatocarcinogenesis. Nutrients.

[48] Yang Q., Liu X., Tang H., Chen Y., Bai L. (2025). Emerging roles of cuproptosis in liver diseases. Dig. Liver Dis..

[49] Ullah M.I., Alameen A.A.M., Al-Oanzi Z.H., Eltayeb L.B., Atif M., Munir M.U., Ejaz H. (2023). Biological Role of Zinc in Liver Cirrhosis: An Updated Review. Biomedicines.

[50] Parisse S., Gianoncelli A., Isani G., Gambaro F.L., Andreani G., Malucelli E., Aquilanti G., Carlomagno I., Carletti R., Mischitelli M. (2023). Severity of Hepatocyte Damage and Prognosis in Cirrhotic Patients Correlate with Hepatocyte Magnesium Depletion. Nutrients.

[51] Falcone E., Okafor M., Vitale N., Raibaut L., Sour A., Faller P. (2021). Extracellular Cu2+ pools and their detection: From current knowledge to next-generation probes. Coord. Chem. Rev..

[52] Hara T., Takeda Taki Takagishi T., Fukue K., Kambe T., Fukada T. (2017). Physiological roles of zinc transporters: Molecular and genetic importance in zinc homeostasis. J. Physiol. Sci..

